# Errata

**Published:** 1881-08

**Authors:** 


### ERRATA.

IN the July number of this journal, there occurred some errata in the excellent article of Dr. Fellger, on *Magna est veritas, et prævalebit.* On page 316, for *Magnus*, read *Magna;* at 15th line from bottom for *New Chatel*, read *Neuchatel;* at 3d line from bottom, for *Krichoff & Prunsen*, read *Kirchoff & Bunsen;* on page 318, line 16 from top, the semicolon should be omitted and a period follow, *hyperoxide.* The sentence should read thus: "Ozone possesses a much greater power to oxydize than oxygen, and can turn pure silver into brown silver hyperoxide. Air, which contains in one million parts, one part of ozone, still retains its peculiar odor." Page 317, line 19, for "on life," read "or life." In Dr. Rushmore's article, in the same number, at p. 340, line 6, for "Carbo Veg. relieved," read "Carbo Veg. 900 (Fincke) cured at once."

